# Linking Binary Gene Relationships to Drivers of Renal Cell Carcinoma Reveals Convergent Function in Alternate Tumor Progression Paths

**DOI:** 10.1038/s41598-019-39875-y

**Published:** 2019-02-27

**Authors:** William L. Poehlman, James J. Hsieh, F. Alex Feltus

**Affiliations:** 1 Clemson University Department of Genetics & Biochemistry, Clemson, SC USA; 2 Molecular Oncology, Department of Medicine, Siteman Cancer Center, Washington University, St Louis, MO USA

## Abstract

Renal cell carcinoma (RCC) subtypes are characterized by distinct molecular profiles. Using RNA expression profiles from 1,009 RCC samples, we constructed a condition-annotated gene coexpression network (GCN). The RCC GCN contains binary gene coexpression relationships (edges) specific to conditions including RCC subtype and tumor stage. As an application of this resource, we discovered RCC GCN edges and modules that were associated with genetic lesions in known RCC driver genes, including VHL, a common initiating clear cell RCC (ccRCC) genetic lesion, and PBRM1 and BAP1 which are early genetic lesions in the Braided Cancer River Model (BCRM). Since ccRCC tumors with PBRM1 mutations respond to targeted therapy differently than tumors with BAP1 mutations, we focused on ccRCC-specific edges associated with tumors that exhibit alternate mutation profiles: VHL-PBRM1 or VHL-BAP1. We found specific blends molecular functions associated with these two mutation paths. Despite these mutation-associated edges having unique genes, they were enriched for the same immunological functions suggesting a convergent functional role for alternate gene sets consistent with the BCRM. The condition annotated RCC GCN described herein is a novel data mining resource for the assignment of polygenic biomarkers and their relationships to RCC tumors with specific molecular and mutational profiles.

## Introduction

Renal cell carcinoma (RCC) is a type of cancer that originates from tubular epithelial cells of the kidney. Subtypes of RCC – clear cell, papillary, and chromophobe– demonstrate unique molecular and histological profiles^1^. In recent years, hundreds of RCC tumors from The Cancer Genome Atlas (TCGA^2,3^) and other sources have been deeply analyzed for genes underlying tumor etiology and progression. While many biomarkers have been associated with RCC, there are few causal genes with consistent and stable genetic lesions driving RCC.

In the case of the most common RCC subtype, ccRCC, several biomarkers have been discovered with variable prevalence between individual tumors. The VHL gene is a common initiating mutation, leading to an accumulation of lipids and glycogens in the tissue^4^. Loss of VHL function is insufficient to develop ccRCC. Epigenetic regulators such as PBRM1 and BAP1 – which act as tumor suppressors – are frequently mutated and associated with distinct clinical outcomes in ccRCC patients^5^. Loss of function of another chromatin-modifying gene – KDM5C – is also associated with unique clinical outcome^6^. BAP1 mutations occur at a near mutually exclusive manner from PBRM1 mutations, and tumors respond to standard of care molecularly-targeted drugs differently depending on which mutations they exhibit^6,7^. However, multiple clonal driver subtypes of ccRCC in which BAP1 and PBRM1 mutations co-occur are possible^8^. Other common ccRCC mutations include a histone methyltransferase – SETD2 – and the mTOR kinase which plays key roles in cell growth^9^. These biomarkers are clearly relevant to understanding ccRCC biology, but aberrations in these genes are not always consistent between tumors and probably do not fully explain ccRCC tumor progression.

Biomarker inconsistency, a prime motivation for personalized medicine, can partly be attributed to tumor heterogeneity which is a genotyping challenge given that certain regions of a tumor may contain mutations that are unique from other regions of the tumor. A Braided Cancer River Model (BCRM) has defined stages of mutation accumulation that lead to clear cell RCC (ccRCC)^10^: initiating, early, intermediate, and speedy mutations. A key aspect of this model is that genetic pathways can operate in parallel to drive tumorigenesis, suggesting that mutations in different genes at various stages of the model can result in convergent evolution of cancer cells^7,10^. Thus, targeting parallel genetic pathways with similar phenotypic outputs becomes a challenge in treating and preventing cancer. Polygenic biomarker discovery may provide insight on these parallel pathways and suggest possible therapeutic targets. Given that mutations in chromatin-modifying genes will greatly alter mRNA expression levels^4^, identifying RCC-subtype specific gene expression patterns may pave the way for more robust drug targeting.

One method to discover novel biomarkers is through gene coexpression network (GCN) analysis. A GCN is a graph of nodes and edges, where nodes are gene products (e.g. mRNA) and edges are binary relationships between genes (e.g. Spearman correlation). A network of significant edges can be extracted using random matrix theory (RMT)^11,12^ or a via soft thresholding to identify functional modules as per WGCNA^13^. Gene modules of tightly connected nodes are partitioned from the GCN using techniques such as link communities^14^. Modules are then tested for enrichment in known biochemical activity, allowing inference of novel gene function^15,16^. Knowledge Independent Network Construction (KINC) is a software package that builds GCNs and tracks the conditions under which significant edges exist^17^. Prior to performing correlation analysis for a given gene pair, KINC uses Gaussian Mixture Models (GMMs) to detect one or more sample clusters in the pairwise expression data. Each sample cluster in each pairwise gene comparison is tested for correlation. This procedure reduces extrinsic noise due to sample variation, and since the samples are tracked it is possible to test each edge for overrepresentation of an attribute or condition (e.g. sex, tumor subtype, tumor stage). For example, Dunwoodie *et al*. used KINC to identify a gene coexpression module that is specific to glioblastoma, an aggressive form of brain cancer^18^. Thus, KINC is an appropriate method to discover condition specific gene relationships in a complex mixture of gene expression profiles.

The purpose of this study was to use KINC to identify RCC subtype-specific GCN edges. In addition, we searched for GCN edges specific to tumors with co-occurring mutations in known genes relevant to ccRCC. The GCN was constructed from 1,009 RCC RNAseq datasets from TCGA which included the three major RCC subtypes. These datasets span various tumor stages as well as clinical attributes such as gender and vital status. We assigned GCN edges to ccRCC tumor subsets that have accumulated specific sets of known driver mutations.

## Results

**Figure 1:**
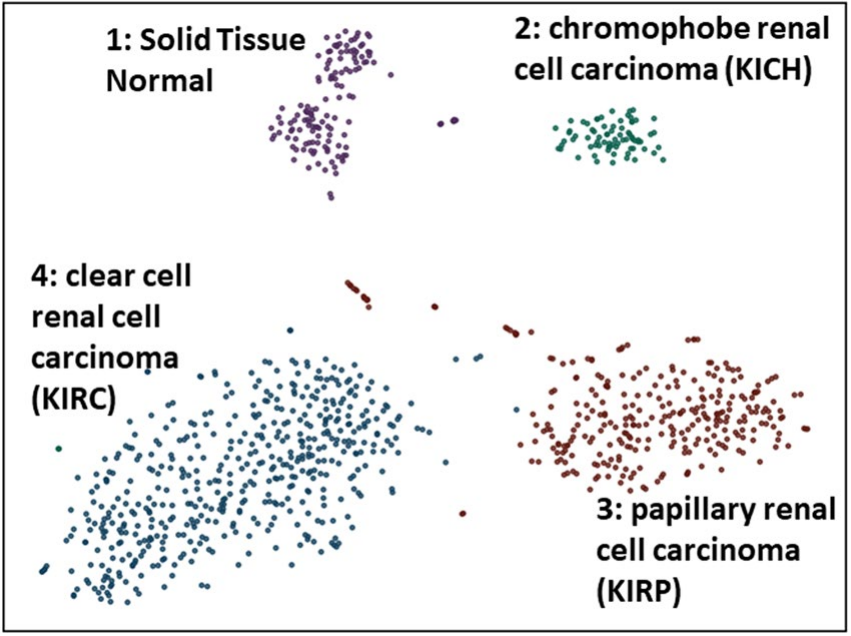
Overview of TCGA RCC Expression Data. A total of 128 “solid tissue normal” kidney samples and 860 “primary tumor” samples with were used in this study. Shown are four consensus clusters each with a unique color identified from 1000 t-SNE runs.

We downloaded and parsed 1,021 gene expression quantification files representing clear cell renal cell carcinoma (KIRC), papillary renal cell carcinoma (KIRP), and chromophobe renal cell carcinoma (KICH) into a 1,021 × 60,483 gene expression matrix (GEM). The GEM contained 860 samples that are annotated for specific tumor stages and 128 samples that are annotated as “Solid Tissue Normal”. In addition, there are 33 primary tumor samples that were not annotated for a specific tumor stage. The matrix was log base 2 transformed and 12 outlier samples were removed. Following quantile normalization of the GEM, we performed 1,000 iterations of t-distributed stochastic neighbor embedding (t-SNE)^19^ and circumscribed clusters using HDBSCAN^20^ and the Cluster Ensembles method^21^ (Fig. 1). Four clusters were identified: Cluster 1 (solid tissue normal enriched; FDR = 4.03E-67); Cluster 2 (KIRP enriched; FDR = 4.88E-83); Cluster 3 (KICH enriched; FDR = 6.84E-40); and Cluster 4 (KIRC enriched; FDR = 5.32E-70). The sample to cluster assignment is available in Supplemental Table 1.

**Figure 2:**
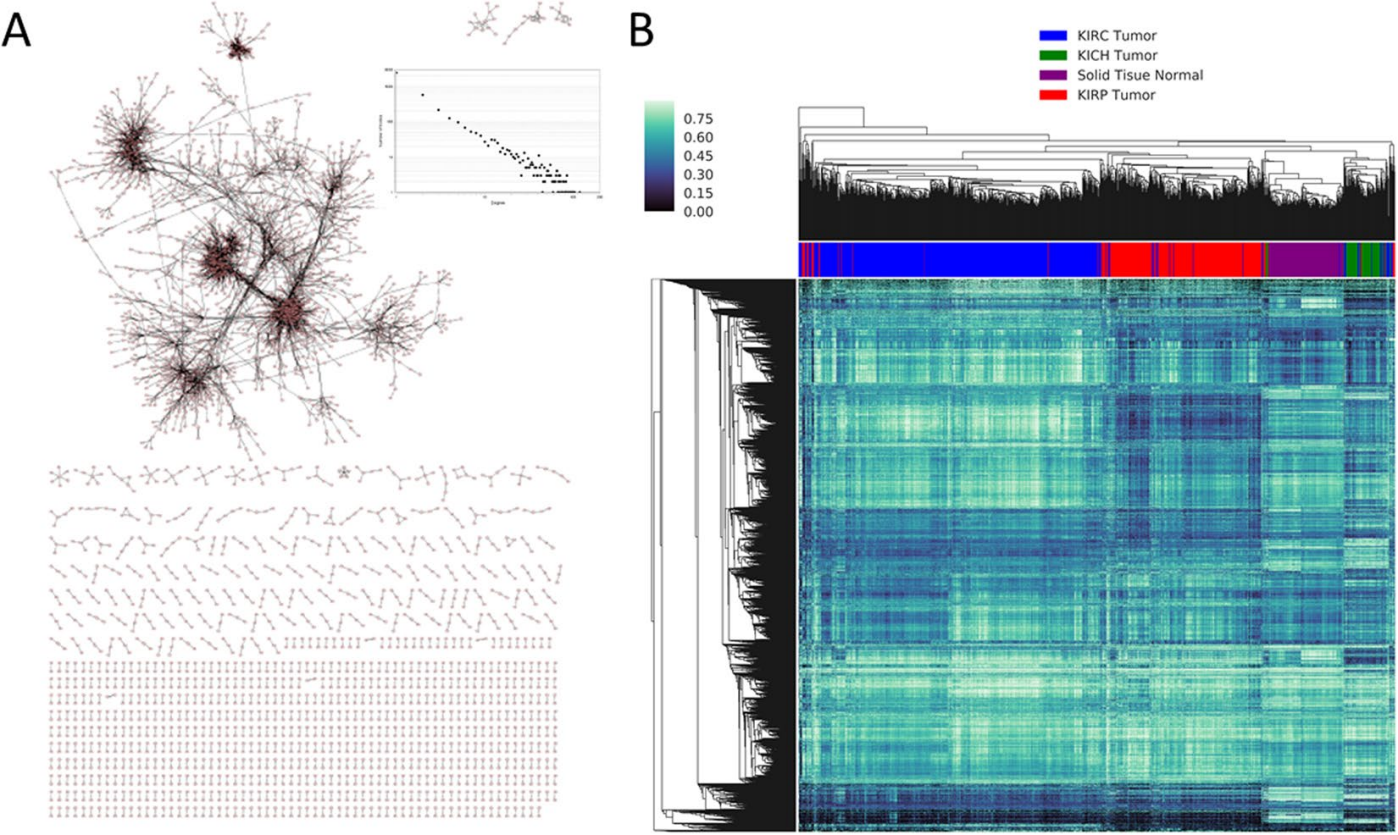
Renal cell carcinoma (RCC) gene coexpression network. (**A**) The RCC GCN demonstrates scale-free topology and contains 4,121 nodes and 10,451 edges. (**B**) A gene expression intensity heatmap of the 4,121 genes is shown.

Using the preprocessed GEM as input, a condition-annotated GCN was constructed using KINC. This RCC GCN contains 4,121 nodes, 10,451 edges, and demonstrates scale-free topology (R2 = 0.933; Fig. 2A). A heatmap presented in Fig. 2B provides a visual overview of expression patterns of these 4,121 genes between the cancer subtypes. Notably, two KIRC cancer subgroups can be seen. The GCN includes edges composed of genes necessary for normal kidney development, such as the Wilms tumor protein (WT1) which was found to be coexpressed with genes such as LMX1B^22^. Edges in the GCN were tested for enrichment of attributes such as cancer type, tissue type, tumor stage, and vital status using a Fisher’s exact test (Table 1). The RCC GCN with enrichment p-values for every edge is available in Supplemental Table 2. Edges that were enriched (adj. p < 0.001) for “Solid Tissue Normal” were extracted to produce a “non-tumor” GCN (Supplemental Table 3). Edges that were enriched for “Primary Tumor” were extracted to produce a “tumor” GCN (Supplemental Table 4). The non-tumor GCN had 1416 nodes and 3605 edges. The tumor GCN had 623 nodes and 2361 edges (Supplemental Fig. 1). The number of condition-enriched edges in each of the three GCNs is shown in Table 1.

**Table 1:** GCN Topology & Attribute-Enriched Edges.

	RCC-GCN	Tumor-GCN	Normal-GCN
*Nodes*	4121	623	1416
*Edges*	10451	2361	3605
<*k>*	5.066	7.576	5.089
*R2*	0.933	0.838	0.850
*Patient KIRC*	6288	1909	2362
*Paitent KIRP*	275	103	50
*Patient KICH*	1807	37	1651
*Primary_Tumor*	2361	2361	0
*Solid_Tissue_Normal*	3605	0	3605
*Tumor_stage_i*	54	16	20
*Tumor_stage_ii*	129	3	100
*Tumor_stage_iii*	432	22	385
*Tumor_stage_iv*	1770	24	1697
*VitalStatus_alive*	9	1	7
*VitalStatus_dead*	2620	280	1987

Link community modules (LCM)^23^ were identified for each GCN (Supplemental Table 5), and functional enrichment tests were performed on each module. Each GCN contains LCMs that were enriched for GO, Reactome, MIM, Pfam, and Interpro annotations. A full list of functionally enriched modules in the RCC GCN is available in Supplemental Table 6. Notably, the non-tumor GCN contains LCM modules that are enriched (Fisher’s Pval < 0.01) for terms related to MET signaling, which is absent in the RCC GCN. The RCC and non-tumor GCN both have modules enriched for VEGF and Notch signaling (Supplemental Tables 7 & 8).

To test if edges where specific to tumors with mutations in known RCC genes, we downloaded somatic mutation profiles for 16 genes that are relevant to RCC^24^ and detected edges that were specific to patients with ccRCC driver mutations. Table 2 presents the number of edges that were specific to patients with mutations in these RCC-associated genes. In order to detect edges relevant to patients with common ccRCC mutation combinations, we performed a Fisher’s exact test for co-occurring VHL and BAP1 mutations (Table 3). In addition, we identified edges in the tumor GCN that are specific to patients with co-occurring VHL and PBRM1 mutations (Table 4).

**Table 2:** GCN Edge-RCC mutation Association.

Mutation	Gene Description	RCC-GCN	Tumor-GCN	Normal-GCN
*VHL*	von Hippel-Lindau tumor suppressor	5282	1755	2330
*PBRM1*	polybromo 1	4254	1362	2274
*SETD2*	SET domain containing 2	265	67	170
*KDM5C*	lysine demethylase 5C	41	33	1
*BAP1*	BRCA1 associated protein 1	41	29	0
*PTEN*	phosphatase and tensin homolog	1	0	0
*MTOR*	mechanistic target of rapamycin kinase	441	31	386
*TP53*	tumor protein p53	154	4	121
*PIK3CA*	PI3-kinase catalytic subunit alpha	3	2	0
*MET*	MET proto-oncogene, RTK	16	1	9
*FAT1*	FAT atypical cadherin 1	0	0	0
*NF2*	neurofibromin 2	2	0	0
*KDM6A*	lysine demethylase 6A	3	0	0
*SMARCB1*	SWI/SNF related	1	0	0
*NFE2L2*	nuclear factor, erythroid 2 like 2	2	0	1
*STAG2*	stromal antigen 2	0	0	0

**Table 3:** KIRC Tumor Edges Associated with Co-Occuring VHL and BAP1 Mutations.

GeneA	GeneB	GeneA Description	GeneB Description	Module	Notes
ENSG00000183918;SH2D1A	ENSG00000181847;TIGIT	SH2 domain containing 1A	T cell immunoreceptor with Ig and ITIM domains	TM0006	^&,‡,†,*^
ENSG00000181847;TIGIT	ENSG00000162739;SLAMF6	T cell immunoreceptor with Ig and ITIM domains	SLAM family member 6	TM0006	^&,‡,†,*^
ENSG00000181847;TIGIT	ENSG00000153283;CD96	T cell immunoreceptor with Ig and ITIM domains	CD96 molecule	TM0006	^&,‡,†,*^
ENSG00000181847;TIGIT	ENSG00000101082;SLA2	T cell immunoreceptor with Ig and ITIM domains	Src like adaptor 2	TM0006	_&,‡,†,*_
ENSG00000198846;TOX	ENSG00000049249;TNFRSF9	thymocyte selection associated high mobility group box	TNF receptor superfamily member 9	NA	^&,‡,†,*^
ENSG00000153563;CD8A	ENSG00000049249;TNFRSF9	CD8a molecule	TNF receptor superfamily member 9	NA	^&,‡,†,*^
ENSG00000163508;EOMES	ENSG00000049249;TNFRSF9	eomesodermin	TNF receptor superfamily member 9	NA	^&,‡,†,*^
ENSG00000181847;TIGIT	ENSG00000089012;SIRPG	T cell immunoreceptor with Ig and ITIM domains	signal regulatory protein gamma	NA	^&,‡,†,*^

^&^Spearman Correlation > 0.80; ^‡^Padj KIRC Patient< 0.001; ^†^Padj Primary Tumor < 0.001; *Padj VHL and BAP1 Mutations < 0.001.

**Table 4:** KIRC Tumor Edges Associated with Co-Occuring VHL and PBRM1 Mutations.

GeneA	GeneB	GeneA Description	GeneB Description	Module	Notes
ENSG00000160185;UBASH3A	ENSG00000153283;CD96	ubiquitin associated and SH3 domain containing A	CD96 molecule	TM0023	^&,‡,†,*^
ENSG00000183918;SH2D1A	ENSG00000160185;UBASH3A	SH2 domain containing 1A	ubiquitin associated and SH3 domain containing A	TM0023	%,‡,†,*
ENSG00000162739;SLAMF6	ENSG00000160185;UBASH3A	SLAM family member 6	ubiquitin associated and SH3 domain containing A	TM0023	^&,‡,†,*^
ENSG00000160185;UBASH3A	ENSG00000101082;SLA2	ubiquitin associated and SH3 domain containing A	Src like adaptor 2	TM0023	^&,‡,†,*^
ENSG00000160185;UBASH3A	ENSG00000116824;CD2	ubiquitin associated and SH3 domain containing A	CD2 molecule	TM0021	^&,‡,†,*^
ENSG00000160185;UBASH3A	ENSG00000089012;SIRPG	ubiquitin associated and SH3 domain containing A	signal regulatory protein gamma	TM0021	^&,‡,†,*^
ENSG00000277734;TRAC	ENSG00000160185;UBASH3A	T cell receptor alpha constant	ubiquitin associated and SH3 domain containing A	TM0021	^&,‡,†,*^
ENSG00000160185;UBASH3A	ENSG00000137078;SIT1	ubiquitin associated and SH3 domain containing A	signaling threshold regulating transmembrane adaptor 1	TM0021	^&,‡,†,*^
ENSG00000160185;UBASH3A	ENSG00000147168;IL2RG	ubiquitin associated and SH3 domain containing A	interleukin 2 receptor subunit gamma	TM0021	^&,‡,†,*^
ENSG00000167286;CD3D	ENSG00000160185;UBASH3A	CD3d molecule	ubiquitin associated and SH3 domain containing A	TM0021	^&,‡,†,*^
ENSG00000182866;LCK	ENSG00000160185;UBASH3A	LCK proto-oncogene, Src family tyrosine kinase	ubiquitin associated and SH3 domain containing A	TM0021	^&,‡,†,*^
ENSG00000198851;CD3E	ENSG00000160185;UBASH3A	CD3e molecule	ubiquitin associated and SH3 domain containing A	TM0021	^&,‡,†,*^
ENSG00000163564;PYHIN1	ENSG00000160185;UBASH3A	pyrin and HIN domain family member 1	ubiquitin associated and SH3 domain containing A	NA	^&,‡,†,*^
ENSG00000231890;DARS-AS1	ENSG00000227191;TCRGC2	DARS antisense RNA 1	T Cell Receptor Gamma Constant 2	NA	^&,‡,†,*^
ENSG00000281881;SPRY4-IT1	ENSG00000109920;FNBP4	SPRY4 intronic transcript 1	formin binding protein 4	NA	^&,‡,†,*^
ENSG00000161405;IKZF3	ENSG00000160185;UBASH3A	IKAROS family zinc finger 3	ubiquitin associated and SH3 domain containing A	NA	^&,‡,†,*^
ENSG00000160185;UBASH3A	ENSG00000143851;PTPN7	ubiquitin associated and SH3 domain containing A	protein tyrosine phosphatase, non-receptor type 7	NA	^&,‡,†,*^
ENSG00000160185;UBASH3A	ENSG00000104814;MAP4K1	ubiquitin associated and SH3 domain containing A	mitogen-activated protein kinase kinase kinase kinase 1	NA	^&,‡,†,*^
ENSG00000160185;UBASH3A	ENSG00000005844;ITGAL	ubiquitin associated and SH3 domain containing A	integrin subunit alpha L	NA	^&,‡,†,*^
ENSG00000263970;RP11-789C17.5	ENSG00000054148;PHPT1	Antisense RNA	phosphohistidine phosphatase 1	NA	^&,‡,†,*^
ENSG00000272505;RP11-981G7.6	ENSG00000253641;LINCR-0001	lincRNA	uncharacterized LINCR-0001	NA	^&,‡,†,*^
ENSG00000234290;AC116366.6	ENSG00000197536;C5orf56	Antisense RNA	chromosome 5 open reading frame 56	NA	^&,‡,†,*^
ENSG00000237721;AF064858.3	ENSG00000235888;AF064858.1	lincRNA	lincRNA	NA	^&,‡,†,*^
ENSG00000231233;CCDC147-AS1	ENSG00000184277;TM2D3	CCDC147 antisense RNA 1	TM2 domain containing 3	NA	^&,‡,†,*^
ENSG00000251320;AC011352.3	ENSG00000248362;AC011352.1	lncRNA	lncRNA	NA	^&,‡,†,*^
ENSG00000218227;RPL19P9	ENSG00000204677;FAM153C	Ribosomal Protein L19 Pseudogene 9	family with sequence similarity 153 member C	NA	^&,‡,†,*^
ENSG00000237471;AC073115.2	ENSG00000229628;AC073115.7	lincRNA	lincRNA	NA	^&,‡,†,*^

^&^Spearman Correlation > 0.80; ^%^Spearman Correlation < −0.80; ^‡^Padj KIRC Patient < 0.001; ^†^Padj Primary Tumor < 0.001; *Padj VHL and PBRM1 Mutations < 0.001.

While some genes are common to the two edge lists in Tables 3 and 4 (CD96, SH2D1A SIRPG, SLA2, SLAMF6), each list contains unique genes that are members of the tumor GCN. Comparing the genes in Table 3 to the genes in Table 4 reveals similar biological function. Enrichment (Fisher’s Pval < 0.001) of GO terms related to T cell activation and immune response are shared between these lists: adaptive immune response (GO:0002250), T cell activation (GO:0042110), positive regulation of natural killer cell mediated cytotoxicity (GO:0045954), and regulation of immune response (GO:0050776).

## Discussion

We constructed a condition-annotated RCC GCN and detected edges that are specific to cancer subtype, tissue type, tumor stage, and unique mutation profile. KINC software allowed us to construct a GCN from diverse kidney cancer samples and identify GCN edges that are specific to only a subset of the input samples. This GCN is a novel data-mining resource for polygenic biomarker assignment to clinically relevant RCC sub-types. To link novel genes to known drivers of ccRCC, we identified 8 edges that are specific to KIRC primary tumors that contain VHL and BAP1 mutations and compared these to 27 edges that are specific to KIRC primary tumors that contain VHL and PBRM1 mutations. These expanded ccRCC driver mutations represent two possible selection routes through the BCRM. Due to a small number of patients containing a combination of VHL, PBRM1, and BAP1 mutations, we were unable to detect edges specific to this multiple clonal driver. We demonstrate that the tumor GCN edges associated with VHL-BAP1 and VHL-PBRM1 mutations contain different genes with similar biological function. Thus, two unique sets of genes can be regulated and selected for in different tumors yielding the same functional result.

Several of the GCN edges associated with mutated gene sets are associated with T cell activation and immune response. The genes in Tables 3 and 4 are both enriched for the following GO ontology terms: adaptive immune response (GO:0002250), T cell activation (GO:0042110), positive regulation of natural killer cell mediated cytotoxicity (GO:0045954), and regulation of immune response (GO:0050776). Identifying ccRCC edges associated with these functions supports the finding of Ricketts *et al*.^24^ that immune signatures related to T cell response are up-regulated in ccRCC compared to other RCC subtypes.

**Figure 3:**
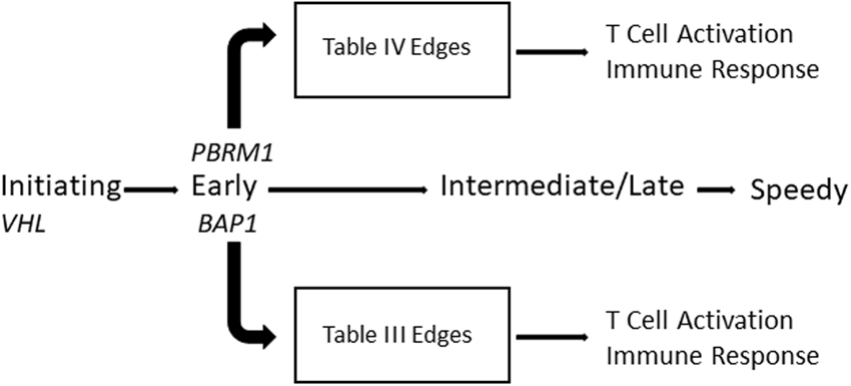
Convergent Gene Coexpression Functions in the Braided Cancer River Model. The Braided Cancer River Model was expanded to include gene coexpression function. GCN edges specific to patients with common ccRCC mutation profiles are enriched for functional annotation terms associated with T cell activation and immune response.

Regardless of whether the patient has co-occurring VHL and BAP1 mutations or co-occurring VHL and PBRM1 mutations, T cell activation genes form coordinated co-expression in the tumor (Fig. 3). It has been shown that T cell exhaustion occurs when T cells are chronically activated due to infection or inflammation^25^. Over time, the T cells lose their function due to increased expression of inhibitory receptors^26,27^. We present binary gene relationships in Table 3 that have been characterized for their role in T cell exhaustion in cancer. TIGIT is an inhibitory receptor that is expressed on the surface of T cells and is associated with poor prognosis in melanoma patients^26,28^. TIGIT is often co-expressed with LAG3, an inhibitory receptor that migrates to the surface of T cells during chronic inflammation, contributing to T cell dysfunction^26,29^. While LAG3 is not present in Tables 3 or 4, we detected seven KIRC-specific edges that contain LAG3 (Supplemental Table 2), implicating this gene in ccRCC regardless of tumor mutation path. We also found TIGIT to be coexpressed with SH2D1A and SLAMF6 in Table 3, which are coexpressed with UBASH3A in Table 4. SH2D1A is a lymphocyte-activating protein that interacts with SLAMF6 to stimulate natural killer (NK) and T cell activity^30–32^. SLA2 — a transcription factor that controls expression of genes that regulate T cell development^33^ — is also present in Tables 3 and 4.

Further, Table 4 contains unique cancer biomarkers that are involved in T cell function. LCK is a tyrosine kinase that functions in normal T-cell development. When this gene becomes mutated and the protein becomes overexpressed, it becomes a proto-oncogene by promoting cellular proliferation and immortality^34^. UBASH3A is a T-cell ubiquitin ligand protein that disrupts T cell receptor signaling by promoting accumulation of inhibitory receptors and T cell apoptosis under certain conditions^35^. Overexpression of UBASH3A is associated with poor prognosis in metastatic breast cancer^36^, and the gene is also associated with autoimmune disorders such as Lupus Erythematosus^37^. UBASH3A is present in 14 of the 27 edges in Table 4, highlighting its importance in ccRCC patients with co-occurring VHL and PBRM1 mutations. It is coexpressed with CD96, an immune checkpoint receptor that plays inhibitory roles in NK cell activity^38^. As we found in Table 3, CD96 is expressed on the surface of T cells with TIGIT, which has also demonstrated inhibitory effects on NK cell function in addition to contributing to T cell exhaustion^39,40^. We also found UBASH3A to be coexpressed with a surface antigen expressed on the surface of T cells, CD2, which has been found to play key roles in NK cell stimulation^41^. Other T cell receptor proteins that we found to be coexpressed with UBASH3A include CD3D and CD3E, which play positive roles in lymphocyte production^42^. The tumorigenic role of UBASH3A should be further explored given its dominant presence in the edges of Table 4. Given that different sets of mutations are associated with unique edges in Tables 3 and 4 that are related to T cell function, we have extended the BCRM to include GCN edges that demonstrate convergent function (Fig. 3).

While we discovered specific edges that contain markers of T cell exhaustion, further studies are needed to understand how these functional clues relate to RCC biology. Because the samples analyzed in this study contained a mixture of T cells and tumor cells, it is not possible to know which cell types produced this result. Computational techniques such as gene set enrichment analysis of marker genes and deconvolution analysis could be used to quantify cell-type composition from gene expression data^43^. Intriguingly, functionally significant interactions between immune cell types have been demonstrated in various cancer types^44^. Thus, the edges described in this report could represent interactions between immune cells and cancer cells in the tumor microenvironment.

Interestingly, Table 4 contains 11 non-coding RNA genes: DARS-AS1, RP11-789C17.5, AC116366.6, CCDC147-AS1, RP11-981G7.6, AF064858.3, AC073115.2, AF064858.1, AC073115.7, AC011352.3, and AC011352.1. Non-coding RNAs are thought to play key roles in cancer by altering gene expression levels through recruitment of chromatin-modifying enzymes or by directly targeting RNA-binding proteins^45,46^. Notably, the antisense RNA DARS-AS1 was found to be correlated with TCRGC2, a T cell receptor^47^ gene, suggesting that this non-coding RNA might play a role in suppressing healthy T cell function. We also detected four edges: RP11-981G7.6- LINCR-0001, AF064858.3- AF064858.1, AC011352.3- AC011352.1, and AC073115.2- AC073115.7 that are each comprised of two long non-coding RNAs that are correlated with each other. We speculate that these non-coding RNAs are targeting parallel genetic pathways during cancer development as per the BCRM. Identification of similar GCN edges can help tackle the challenge of tumor heterogeneity by identifying novel genes and pathways that synchronously contribute to the hallmarks of cancer.

The condition-annotated GCNs described in this report provide a novel data-mining resource for discovering polygenic biomarkers of RCC. By linking edges to mutations in specific genes, we provide a framework for identifying edges that are relevant to specific clinical subtypes of RCC. In addition, this provides a resource for patients who may have genotyped tumors – but no RNA expression data — to link somatic mutations with therapeutic targets developed from genes in this GCN. Interestingly, the non-tumor GCN is larger than the tumor GCN and has a larger number of condition-specific edges. It is possible that accumulation of driver mutations in the tumor lead to gene expression changes in adjacent normal tissue through epigenetic effects. These gene expression changes may lead to metastasis, tumor growth, or recurrence. Thus, in addition to edges in the tumor GCN, edges in the non-tumor GCN may be important biomarkers or potential therapeutic targets.

While this report focused on edges associated with ccRCC driver mutations, the ccRCC-specific edges that were not mutation-associated are worthy of further exploration. For example, one could model these edges in the context of tumor stages as a “time-series” to identify GCN edge patterns acquired or lost during tumor development. With genome-wide mutation profiles, a deeper analysis could test for edge associations beyond the handful of known mutation drivers examined in this report. Finally, our GCN analysis focused on ccRCC but is applicable to other RCC subtypes. We detected 103 edges that are specific to KIRP tumors and 37 edges that are specific to KICH tumors. We suspect that fewer edges were detected for these RCC subtypes due to the smaller number of available TCGA samples relative to ccRCC patients. Regardless, exploration of these additional binary biomarkers is a valuable resource for characterizing the differential molecular and histological presentation of RCC subtypes.

## Methods

### Input Data and Gene Expression Matrix Construction

All available gene expression quantification (FPKM) files for TCGA-KIRC, TCGA-KIRP, and TCGA-KICH patients were downloaded in May 2018 using the CentOS7 binary distribution of the GDC Data Transfer Tool [https://gdc.cancer.gov/access-data/gdc-data-transfer-tool]. 1,021 samples were downloaded – each containing measurement of 60,483 genes – and aggregated into a gene expression matrix (GEM). The preprocessCore R library was used to preprocess the input GEM^48^. Following log base 2 transformation of the data, outlier samples were detected using a Kolmogorov-Smirnov test (KS Dval > 0.15). A total of 12 outlier samples were removed, and the matrix was quantile normalized to reduce technical noise.

Clinical annotations were downloaded directly from the GDC website [https://portal.gdc.cancer.gov/]. Mutation profiles for 843 RCC patients were downloaded from Supplemental Table 1 of Ricketts *et al*.^24^. This table provides mutation profiles for the 16 genes listed in Table 2. All disruptive mutation types were converted to a simple “Mutation/No Mutation” attribute prior to edge enrichment. In the event that a sample in the RCC GEM was not present in this mutation table, all 16 genes were annotated as “No Mutation”. For co-occurring mutation tests, patients with VHL mutations and mutually exclusive mutations in PBRM1 and BAP1 were assigned the “Mutation” attribute.

### Sample Clustering

One thousand iterations of t-SNE were performed using the parallel Python implementation [https://github.com/DmitryUlyanov/Multicore-TSNE]. A perplexity of 30 was used. Clustering of each embedding was performed using the HDBSCAN Python library [https://pypi.python.org/pypi/hdbscan/]. Consensus clusters were identified using the Cluster_Ensembles Python library[https://pypi.org/project/Cluster_Ensembles/], with a minimum cluster size of 10.

### Gene Co-expression Network Construction

The OSG-KINC workflow [https://github.com/feltus/OSG-KINC]^49^ was utilized to execute 50,000 KINC similarity jobs on the Open Science Grid with the following arguments: ‘*./kinc similarity–method sc–clustering mixmod–criterion ICL–min_obs 30–th 0*’. Output was transferred to Clemson University’s Palmetto Cluster and uncompressed. KINC threshold was executing using the following arguments: ‘./*kinc threshold–min_csize 30–clustering mixmod–method sc–th_method sc–th 0.95–max_modes 5*’. A significance threshold of 0.819100 was identified and the GCN was extracted using the following KINC extract arguments: ‘*./kinc extract–min_csize 30–clustering mixmod–method sc–th_method sc–th 0.819100–max_modes 5*’. A representative GCN edge can be found in Supplemental Fig. 2.

### Edge Enrichment Analysis

Edge enrichment for mutations and clinical attributes was performed using the KINC.R package [https://github.com/SystemsGenetics/KINC.R]. Mutations were coded as present or absent in a tumor according to annotations found in^24^. For co-occurring mutation enrichment, a “Mutation” tumor had to have both VHL-PBRM1 (but no BAP1) or VHL-BAP1 (but no PBRM1) mutations. A Fisher’s exact test with a Hochberg p-value correction was used as the default arguments to the *analyzeNetCat* function. Edges were considered to be significantly enriched for a given attribute or set of attributes if the adjusted p value was less than 0.001. Due to the low number of tumors with co-occurring mutation groups (106 VHL/PBRM1, 28 VHL/BAP1), only edges with a cluster size of 250 or smaller were considered for Tables 3 and 4.

### Module Detection and Enrichment Analysis

Link Community Modules^50^ were detected using the linkcomm R package^23^. The “single” hcmethod was used with a minimum module size of 3 edges. Functional enrichment of LCM modules as performed using the FUNC-E package [https://github.com/SystemsGenetics/FUNC-E], which uses a Fisher’s exact test similar to the David method of functional enrichment^51^. For cross-module comparisons, enriched terms were considered significant if the Fisher’s P value was less than 0.001.

## Supplementary information

Supplemental Figures

Supplemental Tables

## Data Availability

All raw data is available from The Cancer Genome Atlas. Analyzed data including networks are available in Supplementary Information.
