# Supplementary material for: Linking Binary Gene Relationships to Drivers of Renal Cell Carcinoma Reveals Convergent Function in Alternate Tumor Progression Paths
Source: Sci Rep. 2019 Feb 27;9:2899. doi: 10.1038/s41598-019-39875-y (PMC6393532; doi:10.1038/s41598-019-39875-y)
Supplement: Supplementary file 1 — Supplemental Figures [file 41598_2019_39875_MOESM1_ESM.pdf]

Linking Binary Gene Relationships to Drivers of Renal Cell Carcinoma Reveals Convergent Function in Alternate Tumor Progression Paths

William L. Poehlman, James J. Hsieh, and F. Alex Feltus

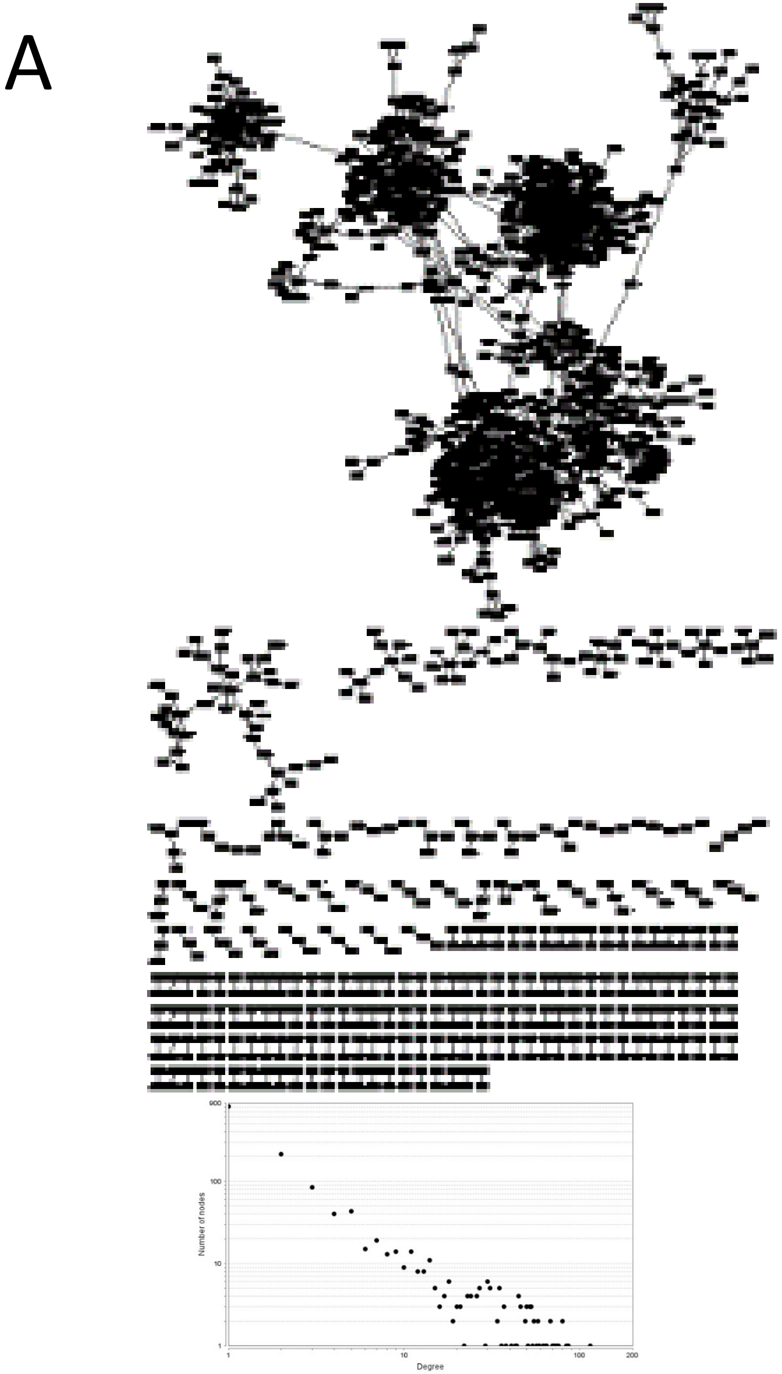

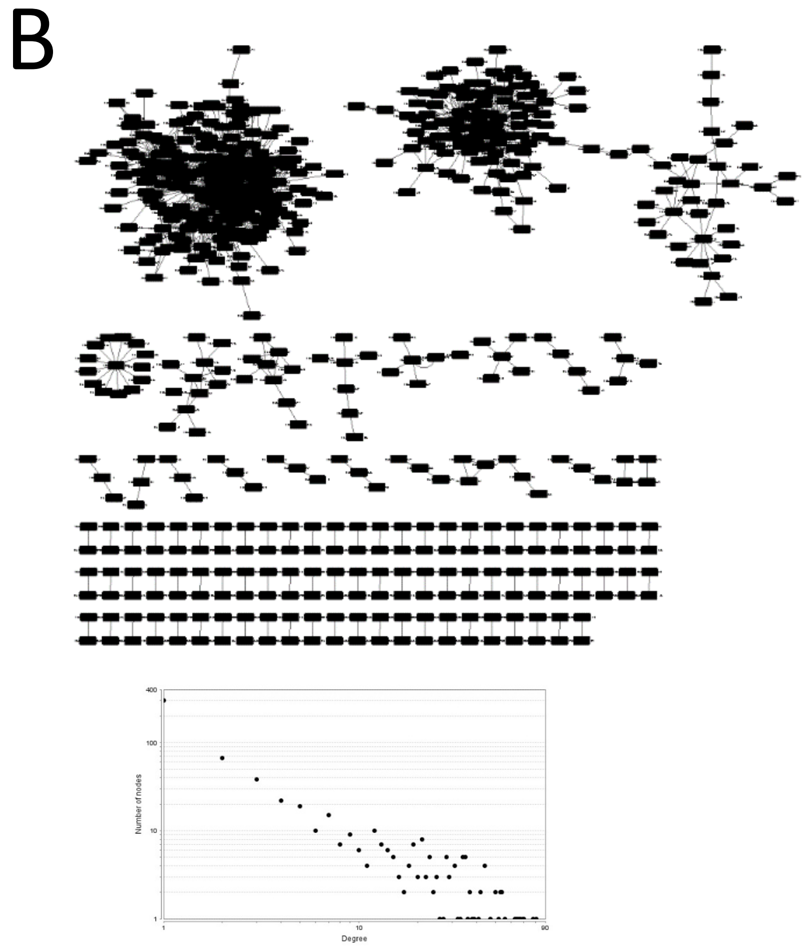

Supplemental Figure 1. Non-tumor and tumor GCNs.

A. Edges enriched for the attribute “Solid Tissue Normal” comprise the non-tumor GCN. **B.** Edges enriched for the attribute “Primary Tumor” comprise the tumor GCN. Both networks demonstrate scale-free topology.

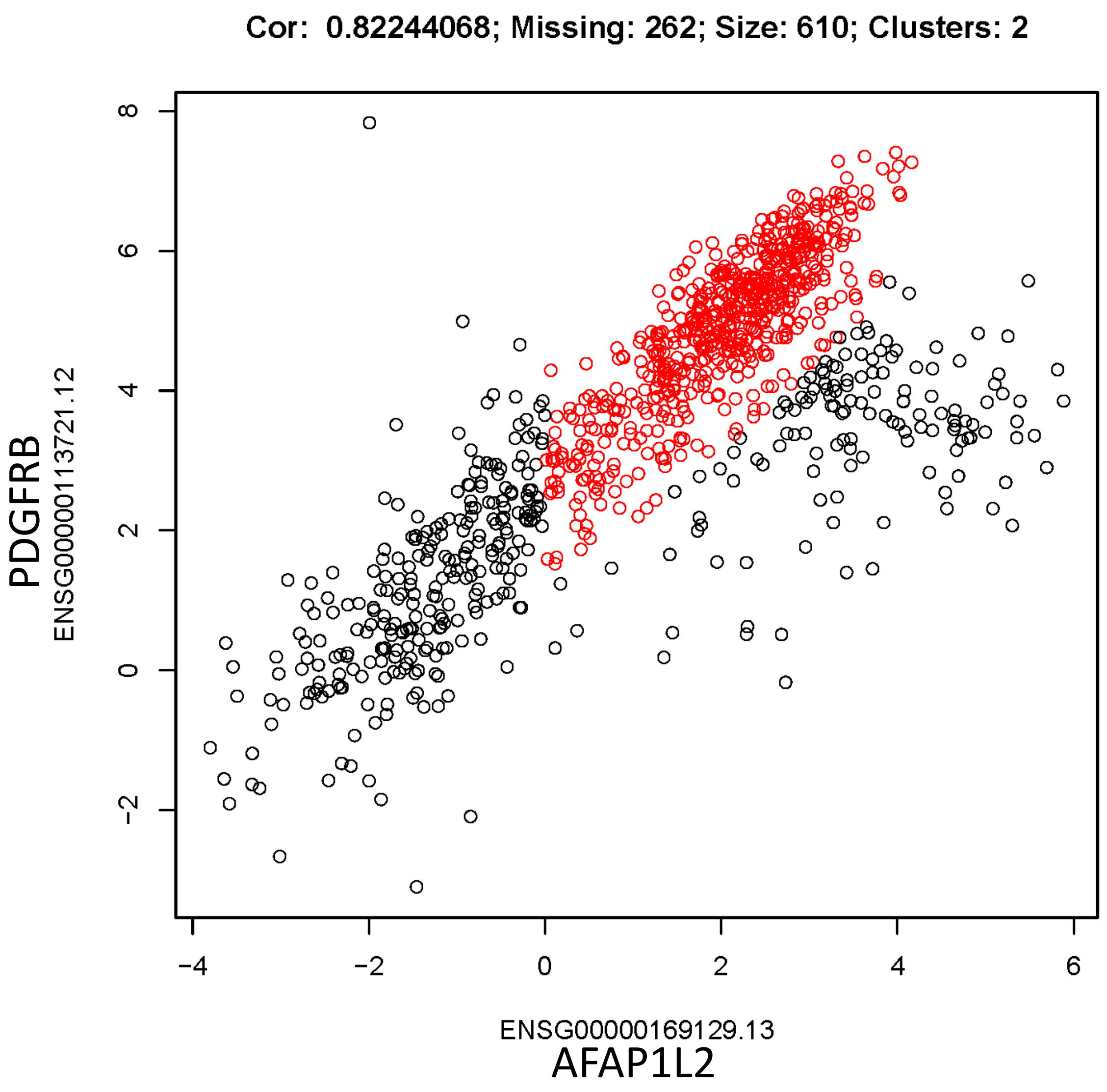

1910111119111119911900011119919111011111111111911019911999
1919199019100991101911111111111101111111911911111099111991
1191110111110101111101119119191110101991199911101199111991
1019199111111019109091911109199191099191111901911111111199
0991101091001111111191190119919919191911019911119111110110
1019991919019101910111111991191110199191111111109111900909
1111119111911911119911110919111011019911119919919111119119
9191911191911091119191101111909109110011999919190119911111
9010010110110110019111191111111110919111019911101911111911
1111991100109911110111101090000110191901901101101111110919
9919101111191191111910910111111011199111911910011101111011
1910911111111911119101110101911119101199011111119111911109
9111119191191119111111111911191919991919110911001111190111
1111911111919191111011111991999110911119111111911119119191
1019101119111119111119199091991010190091911919119099090111
1111191119111019119119191919010199099109111119111111111911
9119111990901910199011119111919911191191001091101009101191
10990190100119119109191101991019109999991

Supplemental Figure 2. A representative GCN Edge.

The left panel shows a scatter plot of pairwise genes expression across all RCC samples between two genes: PDGFRB and AFAP1L2. The red dots indicate tumors that were in a single GMM cluster that was tested for correlation. The right sample string shows which samples were tested for correlation. “1” indicates that sample was tested and is a red dot in the scatterplot. “0” and “9” were excluded from the correlation test.
